# Neurochemical Analysis of Primary Motor Cortex in Chronic Low Back Pain

**DOI:** 10.3390/brainsci2030319

**Published:** 2012-08-21

**Authors:** Neena K. Sharma, William M. Brooks, Anda E. Popescu, Linda VanDillen, Steven Z. George, Kenneth E. McCarson, Byron J. Gajewski, Patrick Gorman, Carmen M. Cirstea

**Affiliations:** 1 Department of Physical Therapy & Rehabilitation Sciences, University of Kansas Medical Center, Kansas City, KS 66160, USA; Email: nsharma@kumc.edu (N.K.S.); pgorman@kumc.edu (P.G.); 2 Department of Neurology, University of Kansas Medical Center, Kansas City, KS 66160, USA; Email: wbrooks@kumc.edu; 3 Hoglund Brain Imaging Center, University of Kansas Medical Center, Kansas City, KS 66160, USA; Email: apopescu@kumc.edu; 4 Program in Physical Therapy, Washington University Medical School, St. Louis, MO 63108, USA; Email: vandillenl@wusm.wustl.edu; 5 Department of Orthopedic Surgery, Washington University Medical School, St. Louis, MO 63108, USA; 6 Department of Physical Therapy, University of Florida, Gainesville, FL 32611, USA; Email: szgeorge@phhp.ufl.edu; 7 Department of Pharmacology, Toxicology & Therapeutics, University of Kansas Medical Center, Kansas City, KS 66160, USA; Email: kmccarso@kumc.edu; 8 Department of Biostatistics, University of Kansas Medical Center, Kansas City, KS 66160, USA; Email: bgajewski@kumc.edu

**Keywords:** chronic low back pain, primary motor cortex, magnetic resonance spectroscopy, *N*-acetylaspartate, myo-inositol

## Abstract

The involvement of the primary motor cortex (M1) in chronic low back pain (LBP) is a relatively new concept. Decreased M1 excitability and an analgesic effect after M1 stimulation have been recently reported. However, the neurochemical changes underlying these functional M1 changes are unknown. The current study investigated whether neurochemicals specific to neurons and glial cells in both right and left M1 are altered. *N*-Acetylaspartate (NAA) and myo-inositol (mI) were measured with proton magnetic resonance spectroscopy in 19 subjects with chronic LBP and 14 healthy controls. We also examined correlations among neurochemicals within and between M1 and relationships between neurochemical concentrations and clinical features of pain. Right M1 NAA was lower in subjects with LBP compared to controls (*p* = 0.008). Left M1 NAA and mI were not significantly different between LBP and control groups. Correlations between neurochemical concentrations across M1s were different between groups (*p* = 0.008). There were no significant correlations between M1 neurochemicals and pain characteristics. These findings provide preliminary evidence of neuronal depression and altered neuronal-glial interactions across M1 in chronic LBP.

## 1. Introduction

Plastic changes have been noted in pain-related brain regions in chronic low back pain (LBP) [1,2,3,4,5]. Increased activity and expansion of the low trunk representation into leg and foot regions in somatosensory cortex (SSC) [1,3] have been reported in LBP. Such changes correlate with pain duration [1]. Moreover, sensorimotor training decreases pain intensity and consequently improves functional outcomes [5]. We recently showed neurochemical changes in SSC in chronic LBP [6]. Specifically, *N*-acetylaspartate (NAA, a marker of neuronal function) measured by proton magnetic resonance spectroscopy (^1^H-MRS) was lower, suggesting neuronal metabolic depression in this area. Further, NAA levels were negatively correlated with pain duration, emphasizing the sensitivity of MRS measurements to capture physiological changes in LBP. Although some evidence suggests that central sensitization and hyperalgesia involve both neurons and glia [7,8,9], conflicting results related to the involvement of glia and their MRS-biomarker, myo-inositol (mI), have been reported [6,10,11] and further investigation is warranted. 

Despite this emerging functional and neurochemical evidence of reorganization in the SSC related to LBP, whether the somatosensory reorganization is accompanied by changes in the primary motor cortex (M1) is not well established. However, recent studies support the concept of M1 reorganization in people with phantom limb pain [12,13], complex regional pain syndrome [14], and chronic LBP [4]. Precisely, people with chronic LBP exhibited posterior and lateral shift and expansion of abdominal muscles representation in M1. The location and size of the muscle representation were correlated with delayed onset of abdominal muscles [4] while spatial topography of alpha event-related desynchronization related to altered anticipatory postural control [15]. Further, this reorganization has been reversed with specific motor training [16]. These changes can be viewed in the context of the anatomical structure and pathways of this area. Several studies suggested that pain might be inhibited via cortico-cortical and cortico-spinal projections of M1 [17,18,19]. Moreover, there is some evidence that stimulation of M1 either by direct electrical [20,21,22] or transcranial magnetic stimulation (TMS) [19,23] alleviate pain from various chronic pain conditions, including chronic LBP, although some studies found contradictory results [24]. Finally, decreased corticospinal excitability relates to pain and disability in chronic LBP [25,26]. 

Taken together, these studies indicate that functional changes might take place in both SSC and M1 in chronic pain. As no study to date has acquired neurochemical indices of M1, in the current study, we examined (1) concentration of neurochemicals specific to neurons (NAA) and glial cells (mI) in M1 trunk representation using ^1^H-MRS in people experiencing chronic LBP; (2) correlations among these neurochemicals within and between M1s; and (3) relationships between M1 neurochemicals and clinical measures of pain and disability. Based on our recent findings in SSC [6] and other relevant studies [10,27,28], we hypothesized that people with LBP would have lower NAA and higher mI levels, reflecting neurochemical plasticity, as well as compromised NAA-mI correlations compared to healthy controls, reflecting neuronal-glial alterations in M1 region. Finally, we expected that neurochemical levels would be correlated with clinical features of pain, as reported previously in sensory and affective brain regions involved in pain processing [6,27].

## 2. Results and Discussion

### 2.1. Demographic and Clinical Findings

There were no significant differences in age between healthy and LBP groups (mean ± SD, 44.6 ± 14.7 years *vs*. 46.1 ± 11.3 years, *p* = 0.8). In the LBP group, the mean duration of pain was 8.8 ± 7.2 years, with an average pain intensity of 4.5 ± 1.9 (highest pain intensity of 7.8 ± 1.5, and lowest pain intensity of 2.3 ± 2.3 on Visual Analog Scale, VAS). Nine participants reported constant pain, eight reported intermittent pain, and for two we had no data, but they were referred from a pain management clinic. Pain referral patterns included nine subjects with radiating pain in buttock and/or leg (four left leg) and eight with localized LBP. 

**Table 1:** Clinical scores (mean ± SD) in low back pain (LBP) group.

Tests	Scores
**Pain**	
VAS_ave_	4.5 ± 1.9
SF-MPQ_sensory_	11.8 ± 5.4
SF-MPQ_affective_	1.9 ± 2.2
SF-MPQ_total_	13.1 ± 6.7
SF-MPQ_PPI_	1.8 ± 0.8
**Disability**	
MODS_total_	14.0 ± 10.5
MODS%	29.3 ± 21.1
**Fear of movement**	
FABQ_physical_	12.4 ± 6.4
FABQ_work_	13.1 ± 11.1
FABQ_total_	34.4 ± 22.6
**Depression**	
BDIS	13.1 ± 13.7

Low Back Pain, LBP; Visual Analog Scale, VAS (10 = worse pain); Short Form McGill Pain Questionnaire, SF-MPQ (45 = severe pain); Modified Oswestry Disability Scale, MODS (50 = maximum disability); Fear-Avoidance Belief Questionnaire, FABQ (66 = most fear with physical and work activities); Back Depression Inventory Scale, BDIS (>40 = extreme depression).

Eight participants took regular pain medications (opiate analgesics, e.g., hydrocodone, oxycodone; anticonvulsant, e.g., Neurontin) and four others took nonsteroidal anti-inflammatory drugs on an as needed basis. Fifteen participants were carrying fulltime regular work responsibilities; three were on disability due to back pain and one was retired. The clinical scores of pain, disability, and depression are shown in Table 1. Overall, our subjects suffered long duration of pain symptoms with moderate intensity and perceived fear of movement. Despite the general severity of these symptoms, depression and disability were only mild. Pain features (Short Form McGill Pain Questionnare, SF-MPQ and VAS_ave_) were positively correlated with perceived fear of movement (*r* = 0.46, *p* = 0.05 and *r* = 0.49, *p* = 0.04, respectively), disability (*r* = 0.84, *p* < 0.001; *r* = 0.54, *p* = 0.02), and depression (*r* = 0.68, *p* = 0.002; *r* = 0.51, *p* = 0.03) in this cohort of LBP.

### 2.2. ^1^H-MRS Spectra Quality

There were no group differences in signal-to-noise ratio (SNR, right M1, *p* = 0.2; left M1, *p* = 0.2) or total brain tissue volume (right M1, *p* = 0.3; left M1, *p* = 0.9) in spectroscopic voxels between groups. Due to poor SNR, we excluded data from one subject’s right hemisphere and two other subjects’ left hemisphere from the LBP group.

### 2.3. Neurochemical Concentrations

**Figure 1:**
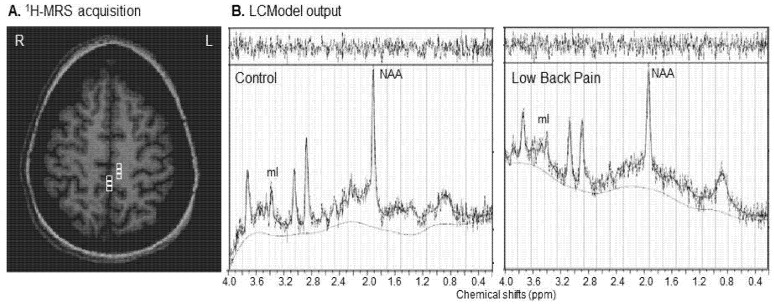
(**A**) Magnetic resonance spectroscopy acquisition: white squares represent the spectroscopic voxels selected in the trunk representation in each primary motor cortex; (**B**) LCModel output showing *N*-acetylaspartate (NAA) and myo-inositol (mI) peaks from right M1 in one representative control and low back pain subject. Lower NAA (8.5 mM *vs*. 9.8 mM) is visible in patient compared to control; ppm, parts per million; R, right; L, left.

**Figure 2:**
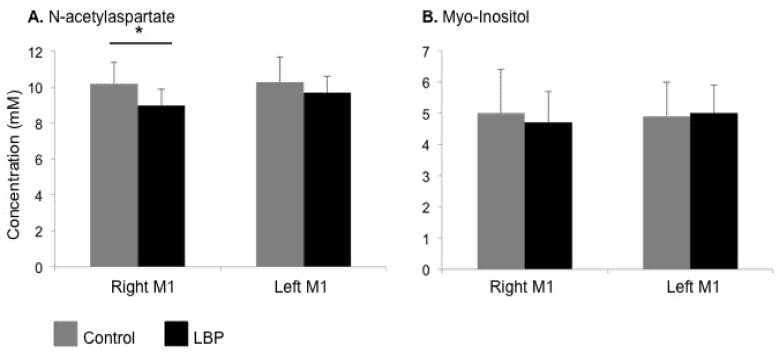
Mean (+SD) concentrations of *N*-acetylaspartate (**A**) and myo-inositol (**B**) in control (grey bars) and low back pain (LBP, black bars) groups in right and left M1. Significantly lower NAA has been observed in right M1 in LBP; * *p* < 0.05.

Normal distributions for individual neurochemicals were verified with frequency and Q-Q plots (Kolmogorov-Smirnov test). Subjects with chronic LBP displayed lower NAA concentrations in right M1 (9.0 ± 0.9 *vs*. 10.2 ± 1.2 mM, *p* = 0.008) compared to controls. Although lower NAA was found in left M1 it was not significantly different from controls (9.7 ± 0.9 *vs*. 10.3 ± 1.4 mM, *p* = 0.2; Figure 1 and Figure 2A). There were no significant changes in mI in either right or left M1 (4.7 ± 1.0 *vs*. 5.0 ± 1.4 mM, *p* = 0.6; 5.0 ± 0.9 *vs*. 4.9 ± 1.1; *p* = 0.6 respectively; Figure 2B). Subgroup analysis of medicated *vs*. un-medicated LBP subjects showed no statistical differences in NAA or mI concentrations. 

### 2.4. Neurochemical Correlations

The strength of correlations was normally distributed in both groups (Kolmogorov-Smirnov test, healthy controls, *p* = 0.2; chronic LBP, *p* = 0.2). In the control group, NAA and mI were strongly correlated within each M1 (Table 2). All inter-M1 correlations were also strong and statistically significant in controls. In contrast, in the LBP group, most correlations were lower and non-significant, although the correlation for mI between hemispheres reached statistical significance (Table 2). Between-group comparison showed lower intra-M1 and inter-M1 mean correlation coefficient in LBP compared to controls (*t* = 2.31, *p* = 0.008).

**Table 2:** Correlation between *N*-acetylaspartate (NAA) and myo-inositol (mI) within (intra-M1) and between (inter-M1) in control and low back pain (LBP) groups.

	Intra-M1			
	Control		LBP	
	*r*	*p*-value	*r*	*p*-value
NAA_R_–mI_R_	0.72	**0.004**	0.17	0.53
NAA_L_–mI_L_	0.74	**0.002**	−0.14	0.62
	**Inter-M1**			
	Control		LBP	
	*r*	*p*-value	*r*	*p*-value
NAA_R_–NAA_L_	0.66	**0.011**	0.03	0.93
mI_R_–mI_L_	0.90	**0.000**	0.78	**0.001**
NAA_L_–mI_R_	0.60	**0.024**	−0.13	0.65
mI_L_–NAA_R_	0.63	**0.015**	−0.17	0.58

*r*, Pearson correlation coefficient; *p*, corresponding *p*-value; L, left M1; R, right M1.

### 2.5. Correlations between Neurochemical Concentrations and Clinical Scores

Although no correlations between NAA or mI concentrations and clinical measures were found to be significant, we did detect some moderately strong trends between left M1 mI and pain duration, pain intensity, and sensory aspects of the SF-MPQ (*r* = 0.52, *p* = 0.05; *r* = 0.52, *p* = 0.06; and *r* = 0.52, *p* = 0.06, respectively). Left NAA and depression scores were also correlated at moderate strength (*r* = −0.46, *p* = 0.08). 

### 2.6. Discussion

Our results demonstrated lower NAA in the trunk representation areas in right M1, and significantly lower correlations between NAA and mI across M1s in participants with chronic LBP compared to controls. There was some evidence that neurochemicals in the left M1 may be correlated with clinical characteristics of pain and depression. 

The magnitudes of NAA alterations described here (12% lower in right M1 and 6% lower in left M1) is similar to spectroscopic findings in other brain regions involved in pain processing, e.g., dorsolateral prefrontal cortex (6.5% lower) [10,27], anterior insula (4.4% lower) [28], and SSC (right, 6%; left, 9% lower) [6]. The significance of altered NAA in M1 is unclear. Although neurodegeneration is associated with lower NAA in some conditions [29], it is unlikely to explain the current findings since we found no significant differences in brain tissue volume (in spectroscopic voxels) between our groups. Alternatively, several studies have highlighted the dynamic nature of NAA. For example, NAA initially falls immediately after traumatic brain injury but then recovers as cognitive function returns [30]. NAA is also correlated with brain glucose consumption [31], suggesting a metabolic role of NAA. Therefore, lower NAA in M1 might suggest altered neuronal mitochondrial metabolism [29,32], which could result from altered peripheral input from either lower back [33,34] or pain pathway [4,25]. Indeed, alterations in motor behavior such as delayed postural control [33], delayed deep back muscle activation [34], and altered gait [35] are reported in these patients. These changes persist beyond resolution of pain symptoms [33] and may be the result of altered motor output from M1, as suggested from evidence that TMS of M1 reduced pain intensity [19,24]. Conversely, changes in the corticospinal M1 excitability are related to pain and disability in chronic LBP [25,26]. Taken together, pain studies clearly suggest reorganization not only in the somatosensory cortex but also in the motor cortices [4,12,13,14]. The interdependence of these two systems is also supported by our findings, e.g., lower NAA in both SSC [6] and M1 in chronic LBP. Further, lower NAA described here may underlie the functional M1 changes. Future studies could evaluate this relationship. 

We did not find significant correlations between NAA levels in M1 and clinical characteristics of pain. It is possible that a more direct measure of the trunk muscle function, such as changes in muscle strength [36,37], muscle activation [4], or muscle volume [38], could provide a better relationship with neuronal integrity in M1 and should be also considered in future studies. The possible correlation between left NAA and depression scores reported here should be considered with caution and repeated with a large sample size to confirm the reliability of this trend. 

Although mI can be an indicator of glial involvement [7,8,9,39] in chronic pain, previous studies reported conflicting results. Higher but not statistically significant mI in orbitofrontal cortex [10] and thalamus [11] has been reported in chronic pain. We found that mI concentrations were not significantly different in M1. mI is a glial cell marker and an osmolyte [39]. Since glutamate and glutamine, other major brain osmolytes [40] were not significantly increased (data not presented here), the changes in mI are unlikely to be driven by changes in osmolarity. mI levels in left M1 were moderately correlated with pain characteristics, although these correlations did not reach statistical significance. Since the astrocytes release trophic factors promoting neuronal survival, synaptogenesis, and neurogenesis after nervous system damage [11,41] and participate in long-term synaptic plasticity [42,43], we can speculate that mI provides information about a potential role of glial cells in left M1 reorganization in LBP. 

Previous studies have shown strong correlations between neurochemical concentrations in functionally-related regions in healthy brain [44,45,46]. Following injury, this correlational structure was disrupted. For example, Cirstea *et al*. have shown that NAA and mI are highly correlated in the motor cortical network under normal conditions and after subcortical stroke this correlation was diminished [46]. Similarly, correlations are disturbed in chronic LBP [6,27]. For instance, altered neurochemical correlations within DLPFC, anterior cingulate, thalamus [27], and SSC [6] suggest disrupted in neurochemical coupling [27] in brain regions involved in both affective-emotional and sensory-discrimination aspects of pain. In agreement with these findings, we noted significantly lower intra-M1 and inter-M1 neurochemical correlations in our patients compared to controls. Although the significance of lower correlations is not clearly understood, we suggest that the effects of pain are not limited on individual neurochemicals but also on the interactions or “communication” between them. 

Although no previous studies have exclusively examined neurochemicals in M1, Grachev *et al*. [27] reported no such changes in sensorimotor area in chronic LBP. This contrary finding can be explained by methodological differences between studies. We specifically analyzed individual voxels in the trunk representation area in M1 using multi-voxel MR spectroscopy imaging whereas Grachev *et al*. [27] used uni-voxel MRS to examine both M1 and SSC. In addition, we investigated absolute neurochemical concentrations rather than ratios to creatine, which might not be a reliable reference in chronic pain [6,47].

Our study has some limitations that might affect its generalizability. We did not measure the outcomes related to lower trunk area such as trunk muscle strength, activation or volume changes of deep back and abdominal muscles. Such measures may provide better understanding of M1 neural changes and more direct correlations between neurochemical levels and clinical outcomes. Second, our sub-group analysis of pain medication effects on neurochemicals was limited to very small numbers per group. Accordingly, our observation of no significant differences in NAA or mI between medicated and un-medicated groups should be interpreted with caution. Third, this study was restricted to examination of neurochemicals in primary motor cortex and did not include other brain regions involved in pain processing, such as DLPFC, anterior cingulate, and insular cortex. A more widespread study of all areas involved in pain processing might provide a better understanding of their relative contribution in addition to M1 to overall brain plastic changes observed in chronic pain. Finally, due to the point-spread function of ^1^H-MRS acquisition, the effective voxel size is larger than the selected voxel size. Thus, we cannot exclude the possibility that our measurements include more than trunk representation in each M1. 

## 3. Methods

### 3.1. Participants

Participants were 19 patients with chronic LBP (4 men) and 14 healthy controls (3 men). Of these, eight LBP and four control participants participated in our earlier study [6]. All participants gave written informed consent to this study, which was approved by the Human Subjects Committee (Institutional Review Board) at the University of Kansas Medical Center. The inclusion criteria for LBP consisted of: (1) medical diagnosis of chronic LBP (>3 months); (2) average pain intensity of at least 3 on a 0 to 10 pain scale (0 = no pain; 10 = worst pain experienced); (3) age between 21 and 65 years; (4) able to understand simple instructions in English language; and (5) normal T2-weighted MR images. The exclusion criteria were: (1) psychiatric, neurological, and/or neuromuscular pathologies based on self-report; (2) spinal cord compression, tumor or infection determined by neurological examination of myotomes, dermatomes and reflex testing; (3) pain in other major joints; and (4) MRI contraindications. Healthy controls without neurological, musculoskeletal or psychiatric pathologies, MRI contraindications and normal T2-weighted images were included.

A detailed description of the experimental protocol (clinical and MRI/^1^H-MRS measurements) has been reported previously [6,46].

### 3.2. Clinical Measurements

Lumbar spine range of motion and neurological examination were conducted to rule out spinal motor nerve root compression and acute spinal pathologies. Participants completed standard self-reported questionnaires for pain (VAS and SF-MPQ), self-perceived disability (Modified Oswestry Disability Scale, MODS), fear of movement (Fear-Avoidance Belief Questionnaire, FABQ) and depression (Back Depression Inventory Scale, BDIS). The reliability and validity of these questionnaires have been reported previously [48,49,50,51].

### 3.3. MRI and ^1^H-MRS Measurements

Structural and spectroscopic data were acquired at 3 Tesla scanner (Siemens Medical Solutions, Erlangen, Germany). Whole brain 3D T1-weighted MRI (MPRAGE, TR = 2300 ms, TE = 3 ms, FOV = 240 mm, matrix = 256 × 256, resolution = 1 × 1 × 1 mm^3^) scans were conducted to estimate brain tissue volume in spectroscopic voxels. T2-weighted MRI (TR = 4800 ms, TE1/TE2 = 18/106 ms, FOV = 240 mm, matrix size = 256 × 256, slice thickness = 5 mm, no gap) scans were conducted to exclude obvious brain pathology in both groups. 

^1^H-MRS was acquired using a PRESS sequence (TR/TE = 1500/30 ms, matrix size = 16 × 16, slice thickness = 15 mm; FOV = 160 mm^2^, in-plane resolution = 5 × 5 mm^2^, spectral width = 1200 Hz). One spectroscopic imaging slab was selected as superiorly and posteriorly as possible to include our region of interest, M1 (Brodmann area 4). Outer voxel saturation bands (30 mm thick) were placed around and above the slab to minimize possible lipid artifact. Automated, followed by manual, shimming was performed before data acquisition to achieve full-width at half maximum of <20 Hz of the water signal from the entire excitation volume. 

### 3.4. ^1^H-MRS Data Processing

The T1-weighted images were segmented into gray matter (GM), white matter (WM) and cerebrospinal fluid using SPM5 (Welcome Department of Cognitive Neurology, London, UK). LCModel [52] was used to calculate neurochemical concentrations. Custom-designed software (Matlab v7.1, 2005) was used to overlay the LCModel output and the segmented T1-weighted images. Three spectroscopic voxels were selected in the M1 trunk representation (Figure 1), based on the following criteria: (1) total brain volume >75%; (2) a SNR > 10; and (3) Cramer-Rao bounds for each neurochemical < 20%. The M1 trunk representation was selected from midline gray matter. The posterior margin was determined from a line drawn from the central sulcus to brain midline on the axial T1-weighted images. The anterior margin was set as the midline gray matter of pre-central gyrus. Spectroscopic voxels were selected from posterior 50% between the central and pre-central sulci. LCModel neurochemical concentrations were corrected for total brain tissue content within each voxel as follows: c = c_LCModel_ × [1/EF_BT_] where c = corrected concentration; c_LCModel_ = concentration from LCModel output (area under the peak of interest), and EF_BT_ = the estimated brain tissue (GM + WM) fraction. The corrected concentrations of all three voxels were averaged to obtain a concentration for our metabolites of interest, NAA and mI, in each M1. We converted the corrected concentration into molar concentrations (millimoles per kilogram wet weight brain tissue) [38] by using a calibration factor obtained by matching the mean NAA concentration in our controls to the average NAA concentration previously reported in healthy human brain [53,54,55]. 

### 3.5. Statistical Analysis

Mean concentrations of two neurochemicals (NAA and mI) and five clinical scores (VAS, SF-MPQ, MODS, FABQ, BDIS) were calculated in LBP group. Mean neurochemical concentrations were also calculated in control group. Between-group comparisons of neurochemical concentrations were analyzed with independent sample *t*-tests. Pearson correlation analysis was used to examine correlations between neurochemicals within each M1 (intra-M1) and between left and right M1 (inter-M1). Fisher’s Z transformation of the correlation coefficients (*r*) was used to analyze overall correlation coefficient (intra-M1 and inter-M1) in each group (mean z scores) and compared between groups by using independent sample *t*-tests. Pearson correlation coefficients were also used to determine relationships between neurochemicals and clinical scores in LBP group. For all comparisons and correlations, significant values were determined at *p* < 0.05 (SPSS 20.0; SPSS Inc., Chicago, IL) with Bonferroni correction, *i.e.*, for neurochemical concentrations (*p* = 0.05/4 = 0.0125); for neurochemical correlations (*p* = 0.05/6 = 0.008); and for clinical correlations (*p* = 0.05/5 = 0.01). 

## 4. Conclusion

We promote the use of the ^1^H-MRS measurements to improve our understanding of the neural changes underlying chronic LBP. Such an approach may also enable us to better understand therapy-related brain changes in this population resulting in development of more efficient pain therapies.
